# The Effects of PAK-Regulated Tumour Vasculature on Gemcitabine Response of Pancreatic Cancer

**DOI:** 10.3390/cancers17213434

**Published:** 2025-10-26

**Authors:** Arian Ansardamavandi, Chelsea Dumesny, Yi Ma, Li Dong, Sarah Ellis, Ching-Seng Ang, Mehrdad Nikfarjam, Hong He

**Affiliations:** 1 Department of Surgery, Austin Precinct, The University of Melbourne, 145 Studley Rd, Melbourne, VIC 3084, Australia; arian.ansardamavandi@student.unimelb.edu.au (A.A.); watsoncj@unimelb.edu.au (C.D.); y.ma11@student.unimelb.edu.au (Y.M.); m.nikfarjam@unimelb.edu.au (M.N.); 2 Department of General Surgery, Monash Health, Clayton, VIC 3806, Australia; 3 Department of Anatomy & Physiology, The University of Melbourne, Melbourne, VIC 3010, Australia; li.dong@unimelb.edu.au; 4 School of Cancer Medicine, La Trobe University, Melbourne, VIC 3000, Australia; sarah.ellis@onjcri.org.au; 5 Olivia Newton-John Cancer Research Institute, Heidelberg, Melbourne, VIC 3086, Australia; 6 Mass Spectrometry and Proteomics Facility, Bio21 Molecular Science and Biotechnology Institute, The University of Melbourne, Parkville, VIC 3010, Australia; ching-seng.ang@unimelb.edu.au; 7 Department of Hepatopancreatic-Biliary Surgery, Austin Health, 145 Studley Rd, Melbourne, VIC 3084, Australia

**Keywords:** pancreatic ductal adenocarcinoma, P21-activated kinase, vascular normalisation, pericyte coverage, endothelial adhesion molecules, gemcitabine sensitivity, VEGF pathway

## Abstract

**Simple Summary:**

Pancreatic cancer is one of the deadliest cancers, partly because its abnormal blood vessels hinder treatments from reaching the tumour effectively. P21-activated kinase 1 (PAK1) and PAK4 play key roles in tumorigenesis, but their specific effects on tumour blood vessels and treatment response are not well understood. In this study, we used immunocompromised mice models to investigate how knockout PAK1 and/or PAK4 change the structure and function of tumour blood vessels and how these changes affect chemotherapy outcomes. We found that PAK1 knockout reduced tumour growth and new blood vessel formation while improving vessel normalisation, whereas PAK4 knockout increased vessel diameter and sensitised cancer cells to chemotherapy. The double knockout of PAK1 and PAK4 did not inhibit tumour growth significantly, but stimulated vascular normalisation, indicating an outcome balanced between PAK1 and PAK4. These findings indicate that PAK1 and PAK4 differentially regulated tumour vasculature and cancer response to chemotherapy.

**Abstract:**

**Background/Objectives**: The tumour microenvironment in pancreatic ductal adenocarcinoma (PDA) is highly complex, influencing both vascular function and therapy response. P21-activated kinases (PAKs) are key regulators of the cellular and immune system, but the specific roles of PAK1 and PAK4 in pancreatic tumour vasculature and chemotherapy sensitivity are unclear. This study investigated the effects of PAK1 and PAK4 on tumour vasculature and therapeutic response in an immunocompromised mouse model. **Methods**: KPC-derived wild type (WT), PAK1 knockout (KO), PAK4KO, or PAK1&4KO pancreatic cancer cells were injected subcutaneously into SCID mice, followed by gemcitabine treatment. Tumour growth, vessel density, pericyte coverage, and endothelial adhesion molecule expression were analysed by histology and immunostaining. A proteomic study was used to identify protein changes. **Results**: PAK1KO significantly reduced tumour growth, enhanced vascular normalisation, upregulated stromal ICAM-1 and VCAM-1, but reduced gemcitabine efficacy. PAK4KO did not inhibit tumour growth but increased vessel diameter and enhanced gemcitabine efficacy. Proteomics study indicated that PAK1KO downregulated proteins involved in the VEGF pathway, while PAK4KO upregulated most proteins involved in the VEGF pathway and downregulated DNA repair proteins, contributing to improved chemotherapy sensitivity. The double knockout of PAK1 and PAK4 did not inhibit tumour growth, although it stimulated vascular normalisation, indicating an outcome balanced between PAK1 and PAK4. **Conclusions**: PAK1 and PAK4 differentially regulated pancreatic tumour vasculature and chemotherapy response. PAK1KO suppressed tumour growth by reducing angiogenesis and enhancing vascular normalisation, whereas PAK4KO enhanced gemcitabine efficacy through vessel dilation.

## 1. Introduction

Pancreatic ductal adenocarcinoma (PDA) is a third cause of cancer mortality in 2025 with an overall 5-year survival of 12% [1,2]. The low survival rate of pancreatic cancer (PC) is mainly due to its aggressive nature and lack of effective treatments, and new therapies are urgently needed [3]. Current treatments for PC, such as FOLFIRINOX and gemcitabine-based combinations, have limited efficacy and high toxicity, highlighting the need for novel therapeutic approaches [4,5]. Late diagnosis, tumour heterogeneity, and intrinsic and acquired resistance mechanisms contribute to the limited efficacy of gemcitabine in PC treatment [6].

The tumour microenvironment of PDA is characterised by a dense stroma and abnormal, dysfunctional blood vessels [7,8]. These structural and vascular barriers severely impair drug delivery and limit therapeutic efficacy [9]. Both immunotherapy and anti-angiogenic therapies, which have shown remarkable success in other malignancies, have achieved only modest benefits in PDA [10,11]. This resistance is largely attributed to the immune-cold tumour microenvironment, extensive stromal fibrosis, and the presence of immunosuppressive myeloid infiltrates [10]. Consequently, conventional anti-angiogenic cancer therapies face challenges, such as resistance, increased tumour invasiveness, and a lack of predictive biomarkers [12]. Vascular normalisation, rather than complete disruption or blockade, has emerged as a promising strategy to enhance anticancer therapy [13,14].

PAKs, a family of serine/threonine kinases, function downstream of Ras-related Rho GTPases and are implicated in cancer [15]. The PAK family, divided into group I (PAK1–3) and group II (PAK4–6), regulates cancer cell migration, invasion, and endothelial permeability through modulation of cytoskeletal dynamics and contractility [16,17]. The immune cells (leukocytes) infiltrating the pancreatic tumour are impaired, which is likely caused by abnormalities in the tumour vascular endothelium, particularly the reduced or altered expression of adhesion molecules such as ICAM-1 and VCAM-1, which are normally required for leukocytes to attach to and cross the vessel wall [18]. Inhibition of PAK1 or PAK4 enhances T cell activation in PC by stimulating anti-tumour immunity through downregulation of PD-L1 [19,20]. Inhibition of PAK4 increased intra-tumoural CD8^+^ T cell infiltration in melanoma and prostate cancer [21,22]. In glioblastoma, targeting PAK4 has been shown to reprogram the vascular microenvironment and improve the efficacy of CAR-T cell immunotherapy [23].

In addition to genetic knockout models, several small-molecule PAK inhibitors—including PF-3758309, FRAX597, and the dual PAK4–NAMPT inhibitor KPT-9274—have shown potent anti-tumour activity in preclinical studies [24,25,26]. Furthermore, advances in targeted protein degradation technologies, such as PROTACs and molecular glues, provide promising strategies to eliminate previously refractory oncogenic drivers [27,28,29]. These pharmacological and degradative approaches may complement genetic knockout studies and enhance the clinical translation of PAK-targeted therapies in PDA.

We have recently reported that knockout of PAK1 or PAK4 enhanced vascular normalisation, stimulating anti-tumour immunity against PC in an immunocompetent syngeneic mouse model [30]. Despite evidence linking PAK signalling to immune regulation and vascular dynamics [14,30], the effects of PAK1 and PAK4 on tumour vasculature and chemotherapy response in PDA remain poorly defined. Elucidating these mechanisms may identify novel therapeutic targets to improve drug delivery and overcome chemoresistance. Immune cells not only shape the tumour immune microenvironment but also regulate angiogenesis and vascular remodelling, thereby influencing tumour growth and metastasis [31]. Our recent work in immunocompetent syngeneic mouse model has shown that PAK1 or PAK4 knockout enhances vascular normalisation and anti-tumour immunity [30]. Here, we used an immunocompromised SCID model to dissect immune-independent isoform-specific effects of PAK1 vs. PAK4 on tumour vasculature and gemcitabine response—focusing on tumour and vascular mechanisms without the interference of immune system effects.

## 2. Materials and Methods

### 2.1. Cell Lines and Cell Culture

The murine PC cell lines used in this study included WT, PAK1KO, and PAK4KO variants. WT and PAK1KO cell lines were originally derived from KPC PAK1^+^/^+^ and KPC PAK1^−^/^−^ mice, as described previously [19]. KPC PAK4KO and KPC PAK1&4KO lines were generated from KPC WT and KPC PAK1KO cells, respectively, using the CRISPR-Cas9 knockout approach detailed in our earlier work [32]. All cell lines were cultured in Dulbecco’s Modified Eagle’s Medium (DMEM) supplemented with 5% fetal bovine serum (FBS) (ThermoFisher Scientific, Melbourne, VIC, Australia) and maintained at 37 °C in a humidified incubator with 5% CO_2_.

### 2.2. Animal Studies

All animal experiments were conducted in accordance with protocols approved by the Austin Health Animal Ethics Committee (A2022-05797 and A2023-05849). Male SCID mice (7 weeks old) were housed in the Austin Health Bioresource Facility under routine health monitoring.

For the bilateral tumour model, KPC WT, PAK1KO, or PAK4KO cells (0.5–1 × 10^6^ cells in 100 μL per mouse) were injected subcutaneously into both flanks. Mice were randomly divided into two groups: one group received gemcitabine treatment, and the other served as untreated controls. Gemcitabine was administered intraperitoneally at 50 mg/kg twice weekly for up to 3 weeks. Tumour growth was measured regularly with a digital calliper, and tumour volume (mm^3^) was calculated using the formula: Volume (V) = Length (L) × Width (W)^2^ × 0.5. At the endpoint, mice were euthanised, tumours excised, and tumour weight (g) recorded.

For the unilateral model, subcutaneous injections of KPC WT or PAK1&4KO cells (0.5–1 × 10^6^ cells in 100 μL per mouse) were administered into a single flank. Mice were observed up to 3 weeks, and this model was used exclusively to assess vascular changes without chemotherapy.

For measuring the diameter of tumour-associated blood vessels, during tumour collection, the blood vessels supporting each tumour were exposed and photographed with a ruler placed alongside for calibration. Vessel diameters were subsequently quantified using ImageJ software (Java v1.8.0_322). The measurement tool was applied by drawing a straight line from the inner edge at the beginning to the end of each vessel cross-section by an independent, blinded observer to the sample groups, providing the diameter in calibrated units. Multiple vessels supplying each tumour were measured, and the mean diameter was used for analysis.

### 2.3. Immunohistochemistry

Formalin-fixed, paraffin-embedded tumour tissues were sectioned at 5 μm using a LEICA RM2245 microtome (Leica Biosystems, Nussloch, Germany). Antigen retrieval was performed in 10 mM Tris-EDTA buffer (pH 9.0) at 99 °C for 30 min, followed by cooling. Endogenous peroxidase was blocked with Dako REAL™ peroxidase solution (S2023, Agilent Technologies, Glostrup, Denmark) for 15 min, and non-specific binding with 5% NGS/1% BSA in TBS-T (Table S1) for 1 h. Sections were incubated overnight at 4 °C with primary antibodies (Table S2), then with goat anti-rabbit HRP polymer (K4003, EnVision+ System-HRP, Dako, Agilent Technologies) for 1 h. Immunoreactivity was visualised using EnVision FLEX DAB+ (K3468, Dako, Agilent Technologies), counterstained with haematoxylin (S3309, Agilent Technologies), mounted in DPX medium (06522, Sigma-Aldrich, St. Louis, MO, USA), and air-dried for 24 h. Whole-slide brightfield images were obtained with the Aperio AT2 scanner (Leica Biosystems), and quantitative analysis was performed with HALO area quantification module v3.0.1 (Indica Labs, Albuquerque, NM, USA) using a constant intensity threshold across all samples.

### 2.4. Immunofluorescence

Following antigen retrieval and blocking, sections were incubated overnight at 4 °C with anti-CD31 antibody, followed by goat anti-rabbit HRP-conjugated secondary antibody for 1 h and tyramide signal amplification with Alexa Fluor™ 488 (B40922, Thermo Fisher Scientific, Waltham, MA, USA) or Alexa Fluor™ 647 (B40926, Thermo Fisher Scientific) for 10 min. After a second retrieval and blocking, sections were incubated overnight with anti-NG2 or anti-α-SMA antibodies (Table S3), then with HRP-conjugated secondary antibody and a second-round fluorophore. Sequential tyramide labelling alternated fluorophores were used to avoid spectral overlap: Alexa Fluor™ 594 (B40925, Thermo Fisher Scientific) was applied second if Alexa Fluor™ 488 was used first, and Alexa Fluor™ 488 was applied second if Alexa Fluor™ 647 was used first. Nuclei were counterstained with DAPI (FP1490, Akoya Biosciences, Marlborough, MA, USA) and mounted in VECTASHIELD antifade medium (H-1700, Vector Laboratories, Burlingame, CA, USA). Whole-slide fluorescence images were acquired with the Zeiss Axioscan 7 (Carl Zeiss AG, Oberkochen, Germany), and quantitative analysis was performed with HALO area quantification FL module v3.0.1, using optimised thresholds for each sample.

### 2.5. Western Blot

WT, PAK1KO, PAK4KO, and PAK1&4KO cells were seeded into 24-well plates and cultured for 48 h. Cells were lysed directly in 2× loading buffer (Table S1). Cell lysates were loaded on 10% SDS-PAGE gels or, for high molecular weight proteins, on gradient gels, and subsequently transferred onto nitrocellulose membranes, which were blocked and incubated with primary antibodies targeting fibronectin, ICAM-1, VCAM-1, PAK1, PAK4, and GAPDH (Table S4). After washing, membranes were exposed to HRP-conjugated goat anti-rabbit IgG secondary antibody (1706515, Bio-Rad, Hercules, CA, USA). Immunoreactive bands were visualised using ECL Select™ chemiluminescent substrate (RPN2235, Cytiva, Amersham, UK) and captured with the ChemiDoc™ MP Imaging System (Bio-Rad Laboratories, Hercules, CA, USA). Band intensities were quantified using ImageJ software (Java v1.8.0_322), and signal values for each target protein were normalised to the corresponding GAPDH control.

### 2.6. Proteomics

KPC WT, PAK1KO, PAK4KO, and PAK1&4KO cells were cultured to ~80% confluency in 10 cm dishes, lysed in RIPA buffer with protease/phosphatase inhibitors (Roche, Mannheim, Germany) (Table S1). Proteins were acetone-precipitated, enzymatically digested, and then analysed by liquid chromatography–mass spectrometry (LC-MS), using data-independent acquisition (DIA), followed by database search using Spectronaut (see Supplementary Materials. Quantification results were then processed in Perseus v2.1.3.0, with LFQ intensities log_2_-transformed and proteins retained if ≥3 valid values per group (4 replicates per group). Differential abundance was assessed by a two-sample Student’s *t*-test, with permutation-based false discovery rate (FDR) truncations (S_0_ = 0.1, FDR < 0.05), and volcano plots were generated. Protein–protein interaction networks were constructed in Cytoscape v3.10.3 using STRING v2.2.0 (Mus musculus, confidence ≥ 0.7), with pathway enrichment analyses performed. Upregulated proteins were visualised in red and downregulated in blue on a continuous scale.

### 2.7. Statistical Analysis

All quantitative results are expressed as mean ± standard error of the mean (SEM). Comparisons between two independent groups were performed using unpaired, two-tailed Student’s *t*-tests under the assumption of Gaussian distribution and equal variance. For datasets containing three or more groups, one-way ANOVA was conducted, and pairwise comparisons among all group means were adjusted using Fisher’s LSD test. When analyses involved two independent variables, a two-way ANOVA was applied with an interaction term, followed by Fisher’s LSD test using a single pooled variance. A 95% confidence interval was applied to all analyses, and statistical significance was defined as *p* < 0.05. Data processing and statistical analyses were carried out using GraphPad Prism software, version 10.5.0 (GraphPad Software, San Diego, CA, USA).

## 3. Results

### 3.1. PAK1 Knockout Reduced Angiogenesis and Decreased the Effect of Gemcitabine on Pancreatic Cancer

**Figure 1:**
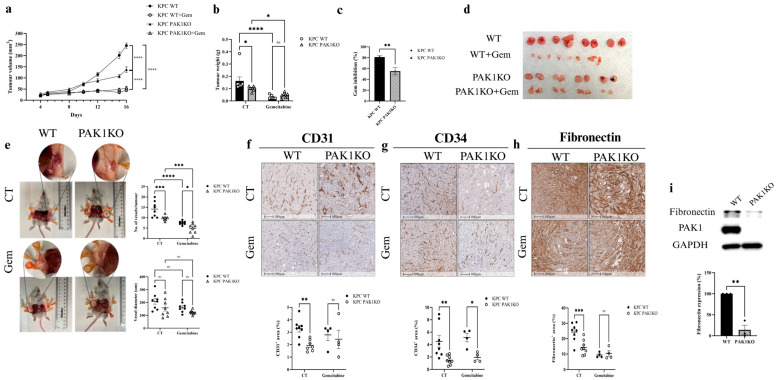
Inhibition of angiogenesis by PAK1KO reduced the effect of gemcitabine on pancreatic cancer. KPC WT and PAK1KO cells were implanted subcutaneously into SCID mice (*n* = 8) and treated ± gemcitabine (50 mg/kg, twice weekly). (**a**–**d**) PAK1KO or gemcitabine reduced tumour growth, but gemcitabine-induced inhibition was attenuated in PAK1KO tumours (**c**). PAK1KO tumours displayed fewer visible vessels without a change in vessel diameter (**e**). PAK1KO reduced the expression of endothelial markers CD31 (**f**) and CD34 (**g**). Fibronectin levels were decreased in PAK1KO tumours (**h**). In vitro analysis confirmed reduced fibronectin production by PAK1KO cancer cells (**i**). WT: wild type; KO: knockout; CT: control; * *p* < 0.05, ** *p* < 0.01, *** *p* < 0.001, **** *p* < 0.0001; ns: not significant. The original Western blot figures can be found in Supplementary File S1.

PAK1 Knockout inhibited xenografted tumour growth of PC (Figure 1a,b) in the immunocompromised SCID mouse model. Gemcitabine suppressed tumour growth in mice inoculated with WT and PAK1KO cells. However, the percentage of gemcitabine inhibition was reduced in the PAK1KO tumour (Figure 1c,d). PAK1KO decreased the number of blood vessels entering the tumour tissues with or without gemcitabine but did not change the vessel diameter (Figure 1e). PAK1 knockout inhibited angiogenesis within the tumour by decreasing the intra-tumoral expression of CD31 (Figure 1f) and CD34 (Figure 1g), two common endothelial cell markers responsible for angiogenesis. Decreased angiogenesis by PAK1KO was associated with reduced fibronectin (Figure 1h). PAK1KO suppressed the expression of fibronectin by PC cells (Figure 1i), contributing to the decreased fibronectin in the PAK1KO tumour. These results indicated that PAK1 inhibited angiogenesis, reducing tumour growth and the inhibitory effect of gemcitabine. Our previous report showed that inhibition of PAK by PF-3758309 (a pan-PAK inhibitor) enhanced the gemcitabine efficacy in a similar SCID mouse model [33]. Here, inhibition of PAK1 by knockout decreased gemcitabine efficacy, suggesting the promoting effect of PF-3758309 is probably via inhibition of PAK4 rather than PAK1. This was confirmed in the following results obtained from PAK4KO.

### 3.2. PAK1 Knockout Upregulated ICAM-1 and VCAM-1 and Promoted Vascular Normalisation

**Figure 2:**
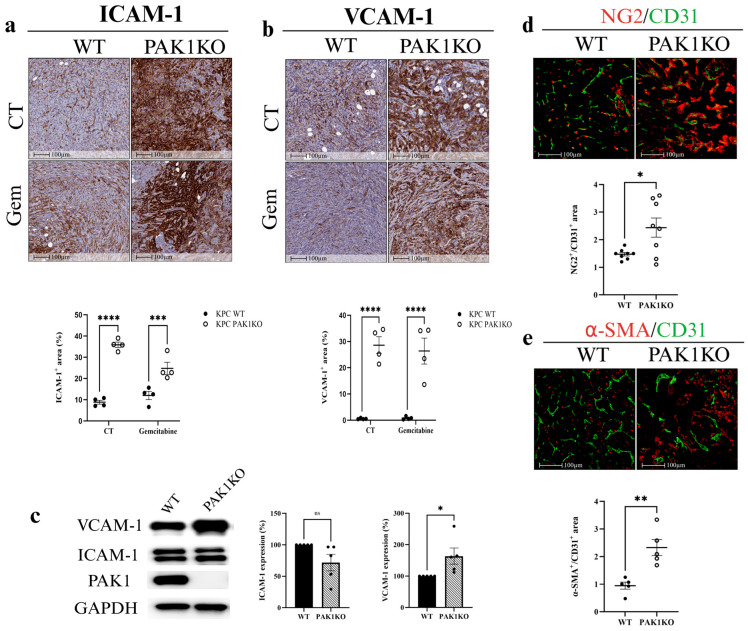
PAK1 knockout upregulated ICAM-1 and VCAM-1 and enhanced vascular normalisation. PAK1KO increased ICAM-1 (**a**) and VCAM-1 (**b**) expression by ~4-fold and ~50-fold, respectively. (**c**) In pancreatic cancer cells, PAK1KO did not affect ICAM-1 but increased VCAM-1 by ~1.5-fold, indicating the stromal origin of tissue upregulation. Vascular normalisation was demonstrated by increased NG2 (red)/CD31 (green) (**d**) and α-SMA (red)/CD31 (green) (**e**) ratios, reflecting enhanced pericyte coverage. WT: wild type; KO: knockout; * *p* < 0.05, ** *p* < 0.01, *** *p* < 0.001, **** *p* < 0.0001; ns: not significant. The original Western blot figures can be found in Supplementary File S1.

Upregulation of endothelial adhesion molecules, including intercellular adhesion molecule-1 (ICAM-1) and vascular cell adhesion molecule-1 (VCAM-1), facilitates intratumoral lymphocyte infiltration, regulating endothelial cell permeability and maturation [34,35,36]. Soluble VCAM-1 also modulates gemcitabine resistance in PC by attracting macrophages to the tumour microenvironment [37]. In tumour tissues, PAK1KO increased the expression of ICAM-1 and VCAM-1 by approximately fourfold and fiftyfold, respectively (Figure 2a,b). In PAK1KO PC cells, the expression of ICAM-1 was not changed, while VCAM-1 was increased by about 150% (Figure 2c), suggesting that the increased ICAM-1 and VCAM-1 in PAK1KO tumour tissues (Figure 2a,b) were predominantly induced in non-tumour stromal cells rather than tumour cells.

Vascular normalisation is characterised by enhanced pericyte coverage, assessed through an increased ratio of mature pericyte markers—NG2 or α-SMA—to the endothelial cell marker CD31 [38]. PAK1KO increased pericyte coverage in pancreatic tumour tissues, as evidenced by higher NG2/CD31 (Figure 2d) and α-SMA/CD31 ratios (Figure 2e). These findings indicated that PAK1 knockout suppressed tumour angiogenesis while promoting vascular normalisation, thereby contributing to the inhibition of pancreatic tumour growth. However, the reduced angiogenesis, rather than the promoted vascular normalisation by PAK1KO, contributed to the decreased effect of gemcitabine on PC growth.

### 3.3. PAK4 Knockout Enhanced the Inhibitory Effects of Gemcitabine by Increasing Blood Vessel Diameter

**Figure 3:**
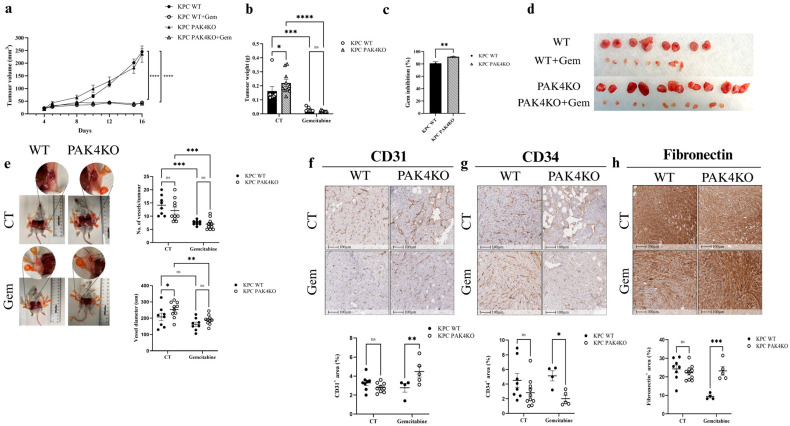
PAK4KO increased the inhibitory effect of gemcitabine by enlarging tumour-associated blood vessel diameter in a SCID mouse pancreatic tumour model. KPC WT and PAK4KO cells were implanted subcutaneously into SCID mice (*n* = 8 WT, *n* = 10 PAK4KO) and treated ± gemcitabine (50 mg/kg, twice weekly). Gemcitabine reduced tumour growth in both groups (**a**,**b**,**d**), with ~10% greater inhibition in PAK4KO tumours (**c**). PAK4KO alone did not change tumour volume (**a**) but modestly increased tumour weight (**b**,**d**). PAK4KO enlarged tumour-associated vessel diameter (**e**). (**f**–**h**) In the absence of gemcitabine, PAK4KO did not alter CD31, CD34, or fibronectin levels. Under gemcitabine, PAK4KO increased CD31 and fibronectin, and reduced CD34. WT: wild type; KO: knockout; CT: control; * *p* < 0.05, ** *p* < 0.01, *** *p* < 0.001, **** *p* < 0.0001; ns: not significant.

PAK4KO did not affect tumour volume (Figure 3a) while increasing tumour weight (Figure 3b) in the immunocompromised SCID mouse model. This was opposite to what occurred in the syngeneic mouse model, where PAK4KO suppressed PC growth [32], indicating that PAK4KO restrained tumour growth by stimulating anti-tumour immunity. Gemcitabine suppressed pancreatic tumour growth in both WT and PAK4KO tumours, demonstrated by reduced tumour volume and weight (Figure 3a,b,d). PAK4KO increased gemcitabine inhibition efficacy by 10% compared to the WT (Figure 3c).

PAK4KO increased the vessel diameter surrounding tumours as measured by ImageJ (Java v1.8.0_322) (Figure 3e). In the absence of gemcitabine, PAK4KO did not affect the expression of CD31 (Figure 3f), CD34 (Figure 3g) or fibronectin (Figure 3h), suggesting that PAK4KO did not affect the angiogenesis within the tumour in this xenograft SCID mouse model. In the presence of gemcitabine, CD31 and fibronectin expression were significantly elevated in the PAK4KO tumour, while CD34 expression was decreased (Figure 3f–h). The elevated levels of CD31 and fibronectin were possibly due to the lower levels of CD31 and fibronectin caused by gemcitabine. The changes in CD31, CD34 and fibronectin by PAK4KO in the presence of gemcitabine seemed to respond to the gemcitabine-induced changes in angiogenesis.

Fibronectin was evaluated as an extracellular matrix protein that supports tumour angiogenesis by promoting endothelial cell adhesion and vessel elongation, rather than as a marker of fibrosis or epithelial–mesenchymal transition [39,40,41]. Reduced fibronectin deposition in PAK1KO tumours corresponded with decreased angiogenesis and vessel density, whereas PAK4KO tumours displayed moderate fibronectin levels associated with enlarged vessel calibre.

**Figure 4:**
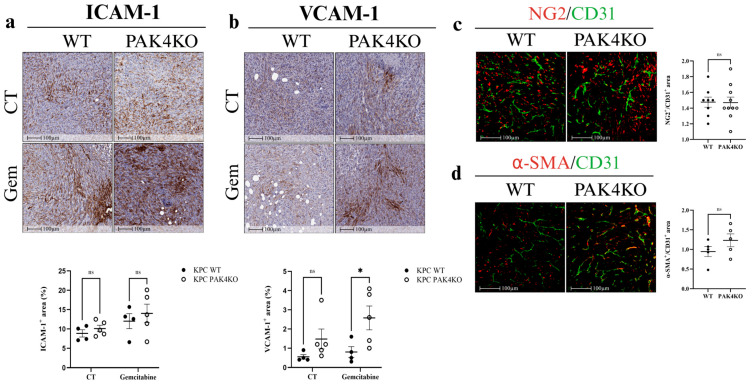
Effect of PAK4KO on ICAM-1, VCAM-1 and vascular normalisation in a SCID mouse pancreatic tumour model. Immunohistochemical analysis of pancreatic tumours showed that PAK4KO did not alter ICAM-1 (**a**) or VCAM-1 (**b**) expression in controls. Vascular normalisation markers, measured as NG2 (red)/CD31 (green) (**c**) and α-SMA (red)/CD31 (green) (**d**) ratios, were unchanged. Under gemcitabine, PAK4KO significantly increased VCAM-1 expression (**b**), while ICAM-1 remained unaffected (**a**). These results indicate that PAK4KO did not intrinsically modulate adhesion molecules or vascular normalisation but enhanced VCAM-1 expression in response to gemcitabine. WT: wild type; KO: knockout; CT: control; * *p* < 0.05; ns: not significant.

In the absence of gemcitabine, PAK4KO did not alter ICAM-1 (Figure 4a) or VCAM-1 (Figure 4b) expression in the control group, nor did it affect vascular normalisation, as indicated by unchanged NG2/CD31 (Figure 4c) and α-SMA/CD31 (Figure 4d) ratios. This was again opposite to that PAK4KO promoted ICAM-1 and VCAM-1 expression and vascular normalisation in a syngeneic mouse model, suggesting the PAK4KO-stimulated anti-tumour immunity may contribute to its effect on vascular normalisation [30]. However, in the gemcitabine-treated group, PAK4KO significantly increased VCAM-1 expression compared with wild-type controls (Figure 4b). These results suggest that PAK4KO does not inherently modulate endothelial adhesion molecules or vascular normalisation but may enhance VCAM-1 expression in response to gemcitabine treatment.

### 3.4. The Effects of Double Knockout of PAK1 and PAK4 on Pancreatic Tumour Growth and Tumour Vasculature

**Figure 5:**
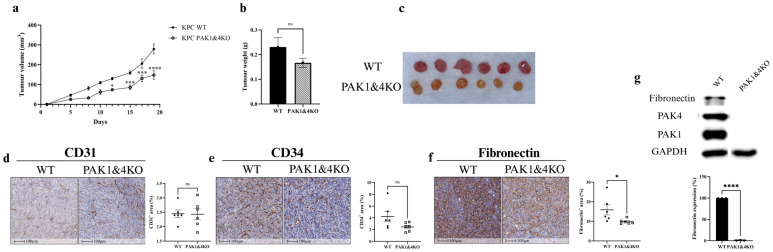
The effects of double knockout of PAK1 and PAK4 on pancreatic tumour growth and angiogenesis in a SCID mouse model. KPC WT (*n* = 6) and PAK1&4KO (*n* = 6) cells were implanted subcutaneously into SCID mice. PAK1&KO significantly reduced tumour volume (**a**), but not tumour weight (**b**), although PAK1&4KO tumours appeared smaller (**c**). CD31 (**d**) and CD34 (**e**) expression were unchanged. (**f**) Fibronectin levels were significantly reduced in PAK1&4KO tumours. (**g**) In vitro analysis confirmed reduced fibronectin expression in PAK1&4KO cells, contributing to the decreased tumour fibronectin production. WT: wild type; KO: knockout; * *p* < 0.05, *** *p* < 0.001, **** *p* < 0.0001; ns: not significant. The original Western blot figures can be found in Supplementary File S1.

PAK1 and PAK4 double knockout (PAK1&4KO) suppressed pancreatic tumour volume (Figure 5a) in a SCID mouse model, but not tumour weight (Figure 5b,c), suggesting that PAK4KO-induced increased tumour weight (Figure 3b) compromised PAK1KO-reduced tumour weight (Figure 1b). The expression of CD31 and CD34 was not changed in the PAK1&4KO tumours (Figure 5d,e), indicating that PAK4KO dominated the effect on angiogenesis and compromised PAK1KO-decreased angiogenesis (Figure 1f,g). Fibronectin levels decreased significantly in double knockout tumours (Figure 5f). PAK1&4KO also suppressed the expression of fibronectin by PC cells (Figure 5g) more dramatically, contributing to overall decreased levels of fibronectin in the PAK1&4KO tumours.

**Figure 6:**
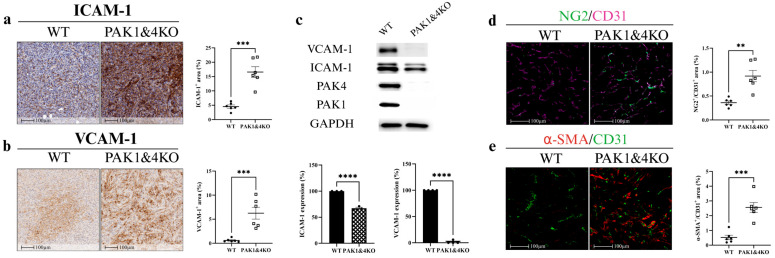
Double knockout of PAK1 and PAK4 upregulated endothelial adhesion molecules and promoted vascular normalisation in pancreatic tumours in a SCID mouse model. Immunohistochemical staining of pancreatic tumours showed that PAK1&4KO increased ICAM-1 (**a**) and VCAM-1 (**b**) expression compared with WT. (**c**) In pancreatic cancer cells, PAK1&4KO reduced ICAM-1 and suppressed VCAM-1 expression, indicating that elevated levels in tumours originate from stromal cells. Vascular normalisation was enhanced, as shown by increased NG2 (green)/CD31 (purple) (**d**) and α-SMA (red)/CD31 (green) ratios (**e**). These findings demonstrate that PAK1&4KO promotes vascular normalisation and enhances stromal expression of endothelial adhesion molecules. WT: wild type; KO: knockout; ** *p* < 0.01, *** *p* < 0.001, **** *p* < 0.0001. The original Western blot figures can be found in Supplementary File S1.

PAK1&4KO markedly increased ICAM-1 (Figure 6a) and VCAM-1 (Figure 6b) expression in pancreatic tumour tissues while decreasing the expression of ICAM-1 and VCAM-1 by cancer cells (Figure 6c), indicating that PAK1&4KO upregulated the ICAM-1 and VCAM-1 in tumour stromal cells rather than the tumour cells. PAK1&4KO enhanced vascular normalisation by promoting the pericyte coverage, demonstrated by increased ratios of NG2/CD31 (Figure 6d) and α-SMA/CD31 (Figure 6e). These findings suggested that PAK1&4KO not only promoted vascular normalisation but also enhanced stromal expression of adhesion molecules, potentially facilitating an immune-permissive tumour microenvironment.

### 3.5. PAK1 and PAK4 Differentially Affected the Molecules Involved in Angiogenesis and Tumour Vasculature

**Figure 7:**
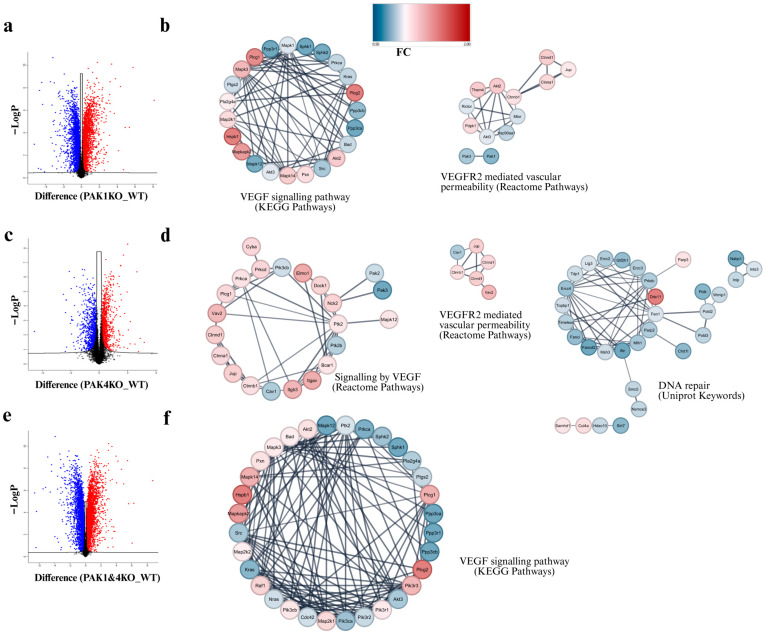
PAK1 and PAK4 differentially affected the molecules involved in angiogenesis and tumour vasculature. Global proteomic profiling compared PAK1KO, PAK4KO, and PAK1&4KO cancer cells with WT, upregulated proteins are depicted in red, and downregulated proteins in blue. (**a**,**c**,**e**) Volcano plots show differentially expressed proteins. (**b**) PPI network analysis indicated alterations in VEGF signalling (KEGG) and VEGFR2-mediated vascular permeability (Reactome) in PAK1KO cells. (**d**) In PAK4KO cells, most proteins involved in VEGF signalling (KEGG) and VEGFR2-mediated vascular permeability (Reactome) were upregulated, while several DNA repair proteins were downregulated, consistent with enhanced gemcitabine response. (**f**) PAK1&4KO cells showed significant alterations in VEGF signalling (KEGG). WT: wild type; KO: knockout.

To explore the molecular changes involved, we conducted an unbiased proteomic study to compare the protein profiles of PAK1KO, PAK4KO, and PAK1&4KO cancer cells with WT cancer cells. The differentially expressed proteins were presented in a volcano plot (Figure 7a,c,e). Protein–protein interaction (PPI) network analysis of the significantly changed global proteomic data indicated significant alterations in the processes involved in the VEGF signalling pathway (KEGG) and VEGFR2-mediated vascular permeability (Reactome). PAK1KO induced downregulation of molecules in the VEGF pathway (Figure 7b) while PAK4KO upregulated the molecules in the VEGF pathway (Figure 7d). The molecules involved in the VEGFR2 pathway were upregulated by both PAK1KO (Figure 7b) and PAK4KO (Figure 7d). PAK4KO also induced downregulation of molecules in the DNA repair capability of cancer cells, which could contribute to stimulating gemcitabine response of cancer cells (Figure 7d). Together, these results suggested that PAK1 and PAK4 differentially affected angiogenesis and tumour vasculature, leading to a differential impact on the effect of gemcitabine in this immunocompromised SCID mouse model. PAK1KO inhibited tumour angiogenesis and promoted vascular normalisation, leading to tumour regression, but reduced the effect of gemcitabine. PAK4KO may stimulate angiogenesis via upregulation of the VEGF signalling pathway (Figure 7), enhancing gemcitabine effect.

Heatmaps of significantly altered proteins were generated to visualise overall expression patterns among WT, PAK1KO, PAK4KO, and PAK1&4KO cells (Figure S1), showing distinct clusters of upregulated and downregulated proteins, illustrating the differential molecular responses to PAK1 and PAK4 knockout.

## 4. Discussion

The individual roles of PAK1 and PAK4 in pancreatic tumour biology are increasingly recognised [42,43], yet their distinct contributions to vascular regulation and chemotherapy response remain underexplored. PAK1 is implicated in cytoskeletal remodelling and endothelial permeability [17], whereas PAK4 influences cancer cell survival, stemness, and therapy resistance [44]. Given the established involvement of PAK signalling in angiogenesis and immune regulation [14], we have further determined the effects of PAK1 and PAK4 on tumour vasculature in PDA, and their impact on therapeutic outcomes.

In the SCID mouse model, PAK1KO or PAK1 and PAK4 double KO promoted vascular normalisation. PAK1KO reduced angiogenesis by inhibiting CD31 and CD34 expression (Figure 1f,g) and decreasing fibronectin levels (Figure 1h,i). Fibronectin not only promotes the differentiation of CD34^+^ cells into endothelial cells in combination with VEGF but also increases its expression around newly developing vasculature, where it plays a critical role in vascular morphogenesis [45,46,47]. Its reduction, together with the decreased CD34, suggests suppression of endothelial differentiation and extracellular matrix (ECM) remodelling required for new vessel formation (Figure 1e–h). While reducing vascular density, PAK1KO increased NG2/CD31 and α-SMA/CD31 ratios (Figure 2d,e), enhancing vascular normalisation [38,48]. Furthermore, PAK1KO modulated the vascular microenvironment and upregulated stromal ICAM-1 and VCAM-1 (Figure 2a,b), consistent with activation of vascular and stromal compartments that could facilitate immune cell adhesion and infiltration in an immune-competent context [49,50,51]. Although direct co-localisation with endothelial markers was not assessed, the higher expression of ICAM-1 and VCAM-1 observed in tumour tissues relative to cultured cancer cells suggests that stromal and endothelial cells likely contribute to their expression within the tumour microenvironment. Nevertheless, cancer cells may also express adhesion molecules in response to tumour microenvironmental cues [52]. Future co-localisation or single-cell analyses are warranted to confirm their precise cellular origin.

PAK4KO did not change vascular density or pericyte coverage (Figure 3f,g and Figure 4c,d) but significantly increased vessel diameter (Figure 3e), contributing to improved drug perfusion, thus increasing gemcitabine efficacy (Figure 3c). Proteomic analysis revealed that most proteins involved in VEGF signalling were upregulated, consistent with increased vessel diameter, alongside downregulation of multiple proteins involved in the DNA repair pathway (Figure 7d). Impaired DNA repair likely increases gemcitabine-induced DNA damage, providing a dual mechanism—vascular and intrinsic—for enhanced chemosensitivity. In this study, fibronectin was analysed as a key extracellular matrix regulator of tumour angiogenesis. Fibronectin deposition facilitates endothelial sprouting and vascular remodelling, contributing to aberrant vessel architecture in PDA [39,40,41]. The reduction in fibronectin observed in PAK1KO tumours aligns with their suppressed angiogenic phenotype, while partial retention in PAK4KO tumours may underlie the observed increase in vessel diameter.

These results indicated that PAK1 and PAK4 differentially regulated tumour vasculature, thus tumour growth and gemcitabine efficacy. In PAK1KO tumours, the overall angiogenic activity was suppressed, whereas vessel maturation increased, indicating a shift toward a more stable but less perfused vascular phenotype. This remodelling limited vascular expansion and nutrient availability, contributing to spontaneous tumour regression in the absence of treatment. However, the same reduction in perfusion restricted gemcitabine distribution, thereby reducing chemotherapy efficacy in the treated group. These findings collectively suggest that PAK1 inhibition induces vascular normalisation characterised by reduced angiogenesis and increased vessel stability, which impacts both tumour progression and drug response. On the other hand, PAK4KO increased vessel diameter, enhancing gemcitabine efficacy but not regressing tumour growth. The data from the PAK1&4KO confirmed these differential roles played by PAK1 and PAK4. PAK1&4KO didn’t regress tumour growth significantly, nor the angiogenesis, suggesting that PAK4KO dominated the vascular regulation and thus tumour growth. PAK1&4KO enhanced vascular normalisation by increasing NG2/CD31 and α-SMA/CD31 ratios (Figure 6d,e) and stimulated stromal ICAM-1 and VCAM-1 expression (Figure 6a,b), indicating that PAK1KO dominated the regulation of vascular normalisation. Nevertheless, PAK1&4KO reduced fibronectin expression (Figure 5f,g), suggesting diminished ECM remodelling associated with vessel sprouting. Although the observed increase in pericyte coverage and ICAM-1 and VCAM-1 expression supports structural vascular normalisation, functional perfusion was not directly assessed. Future studies will incorporate Evans blue dye injection assays to measure vascular perfusion and confirm that the normalised vessels exhibit improved functionality in PAK1KO and PAK4KO tumours [53].

The proteomic data further confirmed the differential roles of PAK1 and PAK4 in regulating tumour vasculature. PAK1KO induced downregulation of molecules involved in the VEGF pathway, while PAK4KO upregulated the molecules in the VEGF signalling pathway (Figure 7). Vasculogenic mimicry (VM), a process whereby cancer cells form vessel-like structures independent of endothelial cells, is increasingly recognised as a contributor to tumour progression and therapeutic resistance in PC [54,55]. Our proteomic data revealed reduced expression of EphA2 and CD34 in PAK1KO and PAK1&4KO cells, two molecules implicated in VM formation and ECM remodelling (Figure S2). EphA2 has been shown to regulate VE-cadherin-mediated signalling and matrix degradation, while CD34 expression has been linked to endothelial-like phenotypes in aggressive cancers [56,57]. The downregulation of these markers suggests that knockout of PAK1 and/or PAK4 may suppress VM formation, thereby contributing to vascular normalisation and altered tumour vascular architecture observed in our models. These findings highlight an additional mechanism through which PAK inhibition may impair tumour adaptability and enhance therapeutic responsiveness. Further study is necessary to confirm the effects of PAKs on VM. The proteomic analysis was performed on pancreatic cancer cell lysates to delineate isoform-specific alterations in intracellular signalling. This analysis revealed pathways related to angiogenesis and DNA repair that may contribute to the observed in vivo vascular and therapeutic phenotypes. A further study is required to validate key angiogenic mediators such as VEGF and VEGFR2 in tumour tissues, particularly in immunocompetent models, to confirm their involvement in PAK-mediated vascular modulation.

Overall, our findings indicated that PAK1KO primarily reduced angiogenesis and promoted vascular normalisation, regressing tumour growth but reducing gemcitabine efficacy, while PAK4KO enlarged vessel diameter, enhancing chemotherapy sensitivity but failing to regress tumour growth. PAK1&4KO maintained the vascular normalisation of PAK1KO, but didn’t inhibit angiogenesis, contributing to the insignificant reduction in tumour weight (Figure 5b). Although our subcutaneous KPC model provided mechanistic insights, it does not fully reflect the therapeutic resistance observed in human PDA. KPC cells are intrinsically gemcitabine-sensitive, while human PDA cell lines such as PANC-1 are markedly resistant (approximately 60 µM) [58,59,60]. A similar study using gemcitabine-resistant human PDA cell, PANC-1 will reveal the effects of PAK inhibition on tumour vasculature and chemotherapy efficacy in gemcitabine-resistant PDA cells. Another limitation of this study is the use of SCID mice, which, while allowing us to isolate vascular effects from adaptive immunity, cannot model the complex stromal–vascular interactions present in orthotopic PDA. Future investigations will incorporate orthotopic, immune-competent, and humanised models to more accurately represent PDA treatment responses.

The present findings highlight the potential clinical relevance of PAK isoform-specific targeting in PDA. PAK1 inhibition promotes vascular normalisation and an immune-permissive tumour microenvironment (TME), whereas PAK4 inhibition enhances gemcitabine response by increasing vessel calibre and downregulating DNA repair pathways. These complementary mechanisms suggest that combined PAK1 and PAK4 inhibition could enhance therapeutic efficacy through both vascular and tumour-intrinsic mechanisms. Preclinical studies have demonstrated that PAK selective inhibitors for PAK1 (FRAX597) and PAK4 (PF-3758309) suppress tumour growth and improve chemotherapy response across multiple solid tumours [24,25]. Moreover, the dual PAK4–NAMPT inhibitor KPT-9274 has entered Phase I clinical evaluation (NCT02702492), showing promising tolerability and target engagement [26]. Given the capacity of PAK inhibition to reprogram the vasculature and modulate immune infiltration, rational combination with gemcitabine or immune checkpoint inhibitors represents a promising translational strategy for future investigation.

## 5. Conclusions

This study identifies distinct roles for PAK1KO and PAK4KO in regulating pancreatic tumour vasculature and chemotherapy response. PAK1 knockout reduced angiogenesis, suppressing tumour growth but decreasing gemcitabine efficacy, while promoting vascular normalisation and endothelial activation. PAK4 knockout increased vessel diameter, enhancing gemcitabine sensitivity, but failed to regress tumour growth. PAK1&4KO didn’t reduce angiogenesis nor tumour growth, but promoted vascular normalisation, indicating the outcomes after balancing of PAK1 and PAK4. These findings highlight the potential for isoform-specific PAK targeting to overcome poor drug delivery and to increase drug efficacy for the treatment of pancreatic cancer.

## Data Availability

Data will be made available upon request from the corresponding author.

## Supplementary Materials

The following supporting information can be downloaded at: https://www.mdpi.com/article/10.3390/cancers17213434/s1, Table S1: Buffers used in methods; Table S2: Primary antibodies for immunohistochemistry. N/A: not applicable; Table S3: Primary antibodies for immunofluorescence. N/A: not applicable; Table S4: Primary antibodies for Western blot. N/A: not applicable; Figure S1: Heatmaps of significantly altered proteins in PAK knockout pancreatic cancer cells. (a) Heatmap showing significantly upregulated and downregulated proteins in PAK1KO compared with WT cells. (b) Heatmap of significantly altered proteins in PAK4KO versus WT cells. (c) Heatmap of significantly altered proteins in double knockout (PAK1&4KO) compared with WT cells. Each heatmap represents the Z-scored log_2_ fold-change values of proteins identified by LC–MS proteomic analysis. Figure S2: PAK1 and PAK1&4 knockout suppress vasculogenic mimicry-associated markers. Proteomic analysis revealed that PAK1KO and PAK1&4KO pancreatic cancer cells exhibited reduced expression of (a) and CD34 (b), suggesting that loss of PAK1 may also impair vasculogenic mimicry. WT: wild type; KO: knockout; * *p* < 0.05, **** *p* < 0.0001; ns: not significant; File S1: The original Western blot figures.

## Abbreviations

The following abbreviations are used in this manuscript:

PDA	Pancreatic ductal adenocarcinoma
PAKs	P21-activated kinases
WT	Wild type
PAK1KO	PAK1 knockout
PC	Pancreatic cancer
DMEM	Dulbecco’s Modified Eagle’s Medium
FBS	Fetal bovine serum
NGS	Normal goat serum
BSA	Bovine serum albumin
CM	Conditioned media
LC	Liquid chromatography
MS	Mass spectrometry
DIA	Data-independent acquisition
ICAM-1	Intercellular adhesion molecule-1
VCAM-1	Vascular cell adhesion molecule-1
PPI	Protein–protein interaction
VM	Vasculogenic mimicry

## References

[1] Siegel R.L., Kratzer T.B., Giaquinto A.N., Sung H., Jemal A. (2025). Cancer statistics, 2025. CA Cancer J. Clin..

[2] Rahib L., Coffin T., Kenner B. (2025). Factors Driving Pancreatic Cancer Survival Rates. Pancreas.

[3] Abbas M., Alqahtani M.S., Alshahrani M.Y., Alabdullh K. (2022). Aggressive and Drug-resistant Pancreatic Cancer: Challenges and Novel Treatment Approaches. Discov. Med..

[4] Adel N. (2019). Current treatment landscape and emerging therapies for pancreatic cancer. Am. J. Manag. Care.

[5] Jamal M.H., Khan M.N. (2025). Developments in pancreatic cancer emerging therapies, diagnostic methods, and epidemiology. Pathol. Res. Pract..

[6] Koltai T., Reshkin S.J., Carvalho T.M., Di Molfetta D., Greco M.R., Alfarouk K.O., Cardone R.A. (2022). Resistance to gemcitabine in pancreatic ductal adenocarcinoma: A physiopathologic and pharmacologic review. Cancers.

[7] Feig C., Gopinathan A., Neesse A., Chan D.S., Cook N., Tuveson D.A. (2012). The pancreas cancer microenvironment. Clin. Cancer Res..

[8] Hessmann E., Buchholz S.M., Demir I.E., Singh S.K., Gress T.M., Ellenrieder V., Neesse A. (2020). Microenvironmental Determinants of Pancreatic Cancer. Physiol. Rev..

[9] Kim S.M., Faix P.H., Schnitzer J.E. (2017). Overcoming key biological barriers to cancer drug delivery and efficacy. J. Control. Release.

[10] Cheung P.F., Lutz M., Siveke J.T. (2018). Immunotherapy and Combination Strategies in Pancreatic Cancer: Current Status and Emerging Trends. Oncol. Res. Treat..

[11] Li S., Xu H.X., Wu C.T., Wang W.Q., Jin W., Gao H.L., Li H., Zhang S.R., Xu J.Z., Qi Z.H. (2019). Angiogenesis in pancreatic cancer: Current research status and clinical implications. Angiogenesis.

[12] Shojaei F. (2012). Anti-angiogenesis therapy in cancer: Current challenges and future perspectives. Cancer Lett..

[13] Shang B., Cao Z., Zhou Q. (2012). Progress in tumor vascular normalization for anticancer therapy: Challenges and perspectives. Front. Med..

[14] Ansardamavandi A., Nikfarjam M., He H. (2023). PAK in Pancreatic Cancer-Associated Vasculature: Implications for Therapeutic Response. Cells.

[15] Rane C.K., Minden A. (2019). P21 activated kinase signaling in cancer. Semin. Cancer Biol..

[16] Whale A., Hashim F.N., Fram S., Jones G.E., Wells C.M. (2011). Signalling to cancer cell invasion through PAK family kinases. Front. Biosci. (Landmark Ed.).

[17] Stockton R.A., Schaefer E., Schwartz M.A. (2004). p21-activated kinase regulates endothelial permeability through modulation of contractility. J. Biol. Chem..

[18] Schmidt J., Mocevicius P., Werner J., Ryschich E. (2012). The role of the tumor endothelium in leukocyte recruitment in pancreatic cancer. Surgery.

[19] Wang K., Zhan Y., Huynh N., Dumesny C., Wang X., Asadi K., Herrmann D., Timpson P., Yang Y., Walsh K. (2020). Inhibition of PAK1 suppresses pancreatic cancer by stimulation of anti-tumour immunity through down-regulation of PD-L1. Cancer Lett..

[20] Naїja A., Merhi M., Inchakalody V., Fernandes Q., Mestiri S., Prabhu K.S., Uddin S., Dermime S. (2021). The role of PAK4 in the immune system and its potential implication in cancer immunotherapy. Cell. Immunol..

[21] Abril-Rodriguez G., Torrejon D.Y., Liu W., Zaretsky J.M., Nowicki T.S., Tsoi J., Puig-Saus C., Baselga-Carretero I., Medina E., Quist M.J. (2020). PAK4 inhibition improves PD-1 blockade immunotherapy. Nat. Cancer.

[22] Abril-Rodriguez G., Torrejon D.Y., Karin D., Campbell K.M., Medina E., Saco J.D., Galvez M., Champhekar A.S., Perez-Garcilazo I., Baselga-Carretero I. (2022). Remodeling of the tumor microenvironment through PAK4 inhibition sensitizes tumors to immune checkpoint blockade. Cancer Res. Commun..

[23] Ma W., Wang Y., Zhang R., Yang F., Zhang D., Huang M., Zhang L., Dorsey J.F., Binder Z.A., O’Rourke D.M. (2021). Targeting PAK4 to reprogram the vascular microenvironment and improve CAR-T immunotherapy for glioblastoma. Nat. Cancer.

[24] Jia X., Zhang J., Pan L., He J., Zhu M., Zhao L., Zhang X., Zhao W., Xie D., Shen X. (2025). Multi-omics analysis reveals RNA polymerase II degradation as a novel mechanism of PF-3758309’s anti-tumor activity. Cell Death Discov..

[25] He H., Dumesny C., Ang C.S., Dong L., Ma Y., Zeng J., Nikfarjam M. (2022). A novel PAK4 inhibitor suppresses pancreatic cancer growth and enhances the inhibitory effect of gemcitabine. Transl. Oncol..

[26] Aboukameel A., Muqbil I., Senapedis W., Baloglu E., Landesman Y., Shacham S., Kauffman M., Philip P.A., Mohammad R.M., Azmi A.S. (2017). Novel p21-Activated Kinase 4 (PAK4) Allosteric Modulators Overcome Drug Resistance and Stemness in Pancreatic Ductal Adenocarcinoma. Mol. Cancer Ther..

[27] Wang C., Zhang Y., Chen W., Wu Y., Xing D. (2024). New-generation advanced PROTACs as potential therapeutic agents in cancer therapy. Mol. Cancer.

[28] Tan X., Huang Z., Pei H., Jia Z., Zheng J. (2024). Molecular glue-mediated targeted protein degradation: A novel strategy in small-molecule drug development. iScience.

[29] Neerasa J., Kim B., Chung H. (2025). Novel dual-targeting PROTAC degraders of GSK-3β and CDK5: A promising approach for pancreatic cancer treatment. Bioorg. Med. Chem..

[30] Ansardamavandi A., Dumesny C., Ma Y., Dong L., Ellis S., Ang C.-S., Nikfarjam M., He H. (2025). P-21 Kinase 1 or 4 Knockout Stimulated Anti-Tumour Immunity Against Pancreatic Cancer by Enhancing Vascular Normalisation. Int. J. Mol. Sci..

[31] Stockmann C., Schadendorf D., Klose R., Helfrich I. (2014). The impact of the immune system on tumor: Angiogenesis and vascular remodeling. Front. Oncol..

[32] Ma Y., Dumesny C., Dong L., Ang C.-S., Asadi K., Zhan Y., Nikfarjam M., He H. (2024). Inhibition of P21-activated kinases 1 and 4 synergistically suppresses the growth of pancreatic cancer by stimulating anti-tumour immunity. Cell Commun. Signal..

[33] Wang K., Huynh N., Wang X., Pajic M., Parkin A., Man J., Baldwin G.S., Nikfarjam M., He H. (2019). PAK inhibition by PF-3758309 enhanced the sensitivity of multiple chemotherapeutic reagents in patient-derived pancreatic cancer cell lines. Am. J. Transl. Res..

[34] Bevilacqua M.P. (1993). Endothelial-leukocyte adhesion molecules. Annu. Rev. Immunol..

[35] Garmy-Susini B., Jin H., Zhu Y., Sung R.-J., Hwang R., Varner J. (2005). Integrin α 4 β 1–VCAM-1–mediated adhesion between endothelial and mural cells is required for blood vessel maturation. J. Clin. Investig..

[36] Williams M.R., Luscinskas F.W. (2011). Leukocyte rolling and adhesion via ICAM-1 signals to endothelial permeability. Focus on “Leukocyte rolling and adhesion both contribute to regulation of microvascular permeability to albumin via ligation of ICAM-1”. Am. J. Physiol. Cell Physiol..

[37] Takahashi R., Ijichi H., Sano M., Miyabayashi K., Mohri D., Kim J., Kimura G., Nakatsuka T., Fujiwara H., Yamamoto K. (2020). Soluble VCAM-1 promotes gemcitabine resistance via macrophage infiltration and predicts therapeutic response in pancreatic cancer. Sci. Rep..

[38] Hamzah J., Jugold M., Kiessling F., Rigby P., Manzur M., Marti H.H., Rabie T., Kaden S., Gröne H.-J., Hämmerling G.J. (2008). Vascular normalization in Rgs5-deficient tumours promotes immune destruction. Nature.

[39] Van Obberghen-Schilling E., Tucker R.P., Saupe F., Gasser I., Cseh B., Orend G. (2011). Fibronectin and tenascin-C: Accomplices in vascular morphogenesis during development and tumor growth. Int. J. Dev. Biol..

[40] Zhou F., Sun J., Ye L., Jiang T., Li W., Su C., Ren S., Wu F., Zhou C., Gao G. (2023). Fibronectin promotes tumor angiogenesis and progression of non-small-cell lung cancer by elevating WISP3 expression via FAK/MAPK/ HIF-1α axis and activating wnt signaling pathway. Exp. Hematol. Oncol..

[41] Topalovski M., Brekken R.A. (2016). Matrix control of pancreatic cancer: New insights into fibronectin signaling. Cancer Lett..

[42] Liu H., Liu K., Dong Z. (2021). The role of p21-activated kinases in cancer and beyond: Where are we heading?. Front. Cell Dev. Biol..

[43] Yeo D., He H., Baldwin G.S., Nikfarjam M. (2015). The role of p21-activated kinases in pancreatic cancer. Pancreas.

[44] Tyagi N., Marimuthu S., Bhardwaj A., Deshmukh S.K., Srivastava S.K., Singh A.P., McClellan S., Carter J.E., Singh S. (2016). p-21 activated kinase 4 (PAK4) maintains stem cell-like phenotypes in pancreatic cancer cells through activation of STAT3 signaling. Cancer Lett..

[45] Nicosia R.F., Bonanno E., Smith M. (1993). Fibronectin promotes the elongation of microvessels during angiogenesis in vitro. J. Cell. Physiol..

[46] Kumar V., Viji R., Kiran M., Sudhakaran P.R. (2012). Angiogenic response of endothelial cells to fibronectin. Biochemical Roles of Eukaryotic Cell Surface Macromolecules: 2011 ISCSM Proceedings.

[47] Wijelath E.S., Rahman S., Murray J., Patel Y., Savidge G., Sobel M. (2004). Fibronectin promotes VEGF-induced CD_34_^+^ cell differentiation into endothelial cells. J. Vasc. Surg..

[48] Qian C., Yang C., Tang Y., Zheng W., Zhou Y., Zhang S., Song M., Cheng P., Wei Z., Zhong C. (2022). Pharmacological manipulation of Ezh2 with salvianolic acid B results in tumor vascular normalization and synergizes with cisplatin and T cell-mediated immunotherapy. Pharmacol. Res..

[49] Singh V., Kaur R., Kumari P., Pasricha C., Singh R. (2023). ICAM-1 and VCAM-1: Gatekeepers in various inflammatory and cardiovascular disorders. Clin. Chim. Acta.

[50] Oppenheimer-Marks N., Davis L.S., Bogue D.T., Ramberg J., Lipsky P.E. (1991). Differential utilization of ICAM-1 and VCAM-1 during the adhesion and transendothelial migration of human T lymphocytes. J. Immunol..

[51] Van Buul J.D., Van Rijssel J., Van Alphen F.P., van Stalborch A.-M., Mul E.P., Hordijk P.L. (2010). ICAM-1 clustering on endothelial cells recruits VCAM-1. BioMed Res. Int..

[52] Harjunpää H., Llort Asens M., Guenther C., Fagerholm S.C. (2019). Cell Adhesion Molecules and Their Roles and Regulation in the Immune and Tumor Microenvironment. Front. Immunol..

[53] Radu M., Chernoff J. (2013). An in vivo assay to test blood vessel permeability. J. Vis. Exp..

[54] Cao Z., Bao M., Miele L., Sarkar F.H., Wang Z., Zhou Q. (2013). Tumour vasculogenic mimicry is associated with poor prognosis of human cancer patients: A systemic review and meta-analysis. Eur. J. Cancer.

[55] Yang J., Liao Y., Mai D., Xie P., Qiang Y., Zheng L., Wang M., Mei Y., Meng D., Xu L. (2016). Tumor vasculogenic mimicry predicts poor prognosis in cancer patients: A meta-analysis. Angiogenesis.

[56] Delgado-Bellido D., Serrano-Saenz S., Fernández-Cortés M., Oliver F.J. (2017). Vasculogenic mimicry signaling revisited: Focus on non-vascular VE-cadherin. Mol. Cancer.

[57] Pisacane A., Picciotto F., Risio M. (2007). CD31 and CD34 expression as immunohistochemical markers of endothelial transdifferentiation in human cutaneous melanoma. Anal. Cell. Pathol..

[58] Zhou J., Wang C.-Y., Liu T., Wu B., Zhou F., Xiong J.-X., Wu H.-S., Tao J., Zhao G., Yang M. (2008). Persistence of side population cells with high drug efflux capacity in pancreatic cancer. World J. Gastroenterol. WJG.

[59] Huanwen W., Zhiyong L., Xiaohua S., Xinyu R., Kai W., Tonghua L. (2009). Intrinsic chemoresistance to gemcitabine is associated with constitutive and laminin-induced phosphorylation of FAK in pancreatic cancer cell lines. Mol. Cancer.

[60] Wang Y., Li F., Luo B., Wang X., Sun H., Liu S., Cui Y., Xu X. (2009). A side population of cells from a human pancreatic carcinoma cell line harbors cancer stem cell characteristics. Neoplasma.

