# Supplementary material for: The Effects of PAK-Regulated Tumour Vasculature on Gemcitabine Response of Pancreatic Cancer
Source: Cancers (Basel). 2025 Oct 26;17(21):3434. doi: 10.3390/cancers17213434 (PMC12607862; doi:10.3390/cancers17213434)
Supplement: Supplementary file 1 [file cancers-17-03434-s001.zip › cancers-3892253-supplementary .pdf]

Supplementary Materials

Materials and Methods

LC MS for proteomic study

LC-MS analysis was performed on an Orbitrap Astral Mass Spectrometer (ThermoFisher Scientific). The LC system was a Vanquish Neo UHPLC (Thermo Scientific) and using the heated trap and elute setup. The trap column was an Acclaim Pepmap nano-trap column (Dionex—C18, 100 Å, 75 µm × 2 cm). The analytical column was a 50 cm µPAC (depth) Neo analytical column. The eluents were water with 0.1% v/v FA (solvent A) and 80% CH3CN with 0.1% v/v FA (solvent B). The flow rate was 300 nl/min and gradient were (i) 0-46 min 1.2-28% B, ii) 46-52 min, 28-50% B (iii) 52-57 min, 50-99% B. Column wash was then activated at 0.8 µL/min (combine flow and pressure control) for 3.1 min followed by trap column wash (combine flow and pressure control with 2 zebra wash cycles at 80% CH3CN) and fast equilibration of the analytical column (combine flow and pressure control). Full MS resolutions were set to 240,000 at m/z 200 and scanning from 380-980 m/z in the profile mode. Full MS AGC target was 500% with a maximum IT of 50 ms. DIA was carried out in the Astral analyser with an isolation window of 2 m/z, normalised HCD collision energy of 25, normalised AGC target of 500% and maximum injection time of 3.5 ms. The cycle time was kept to 0.6 s.

Database search for proteomic study

DIA data were analysed using the direct DIA analysis workflow with default settings on the Spectronaut software (v. 19.9.250324.62635) or DIA-NN (version 1.8.1) and against the reviewed Uniport Homo sapiens database (downloaded Oct 2024). For Spectronaut searches, Trypsin specificity was set to two missed cleavages. Carbamidomethyl (Cys) was defined as a fixed modification. Acetylation (protein N-term), phosphorylation (Ser, Thr and Tyr) and oxidation (Met) as variable modifications. Results are filtered at a Protein and PSM false discovery rate of 1%. Precursor filtering using the Q-value, quantification carried out on the MS2 level and cross-run normalization strategy set to automatic.

Supplementary Tables

Table S1. Buffers used in methods.

Buffers	Content
Tris-EDTA buffer	10 mM Tris base, 1 mM EDTA solution, 0.05% Tween 20, PH 9.0
TBS-T	20 mM Tris-HCl, 137 mM NaCl, 0.1% Tween 20, pH 7.6
2x loading buffer	125mM Tris, 20% glycerol, 4% SDS, 2.5% β-mercaptoethanol, Ph 6.8
Ripa buffer	25mM Tris HCL, 150mM NaCl, 1% Triton X-100, 1% Na deoxycholate, 0.5% SDS, 1mM EGTA, PH 8

Table S2. Primary antibodies for immunohistochemistry. N/A: not applicable.

Protein target	Dilution	Cat. number	Company	Clone name
CD31	1:500-1000	PA5-16301	Invitrogen	N/A
CD31	1:1500	77699	Cell Signaling & Technology	D8V9E
CD34	1:10000-15000	ab81289	Abcam	EP373Y
Fibronectin	1:16000-32000	ab268020	Abcam	EPR23110-46
ICAM-1	1:16000	ab179707	Abcam	N/A
VCAM-1	1:10000	ab134047	Abcam	EPR5047

Table S3. Primary antibodies for immunofluorescence. N/A: not applicable.

Protein target	Dilution	Cat. number	Company	Clone name
CD31	1:500	PA5-16301	Invitrogen	N/A
CD31	1:500-1000	77699	Cell Signaling & Technology	D8V9E
NG2	1:500-1000	AB5320	Merck	N/A
α-SMA	1:5000	14395-1-AP	Proteintech	N/A

Table S4. Primary antibodies for Western blot. N/A: not applicable.

Protein target	Dilution	Cat. number	Company	Clone name
Fibronectin	1:1000	ab268020	Abcam	EPR23110-46
ICAM-1	1:2000	10020-1-AP	Proteintech	N/A
VCAM-1	1:2000	ab134047	Abcam	EPR5047
PAK1	1:2000	2602	Cell Signaling & Technology	N/A
PAK4	1:2000	14685-1-AP	Proteintech	N/A
GAPDH	1:10000	2118	Cell Signaling & Technology	14C10

Supplementary Figures

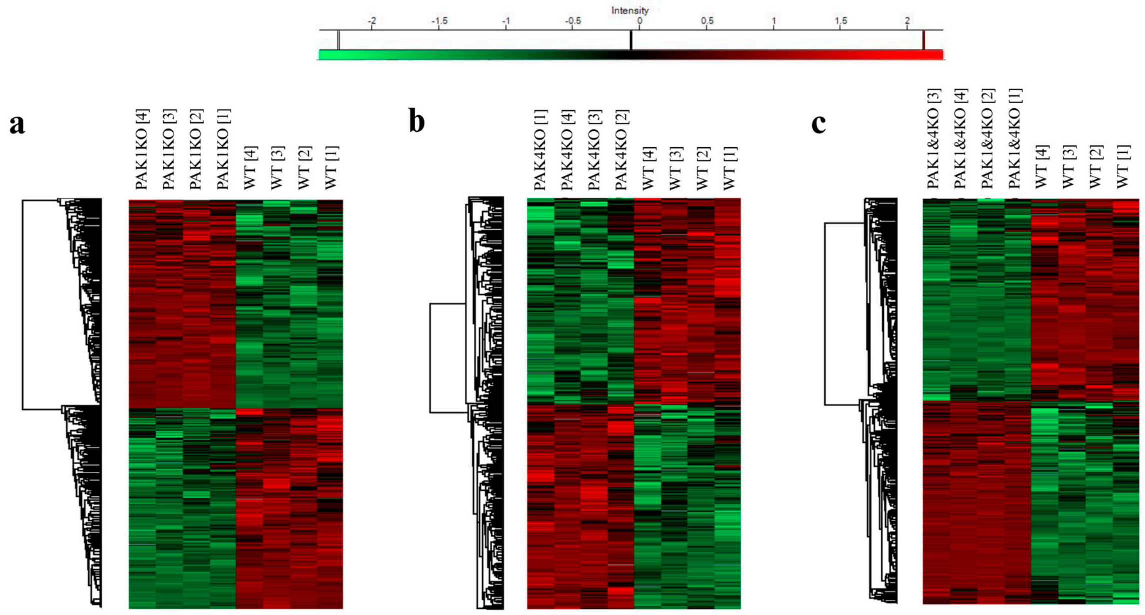

Figure S1. Heatmaps of significantly altered proteins in PAK knockout pancreatic cancer cells. **(a)** Heatmap showing significantly upregulated and downregulated proteins in PAK1KO compared with WT cells. **(b)** Heatmap of significantly altered proteins in PAK4KO versus WT cells. **(c)** Heatmap of significantly altered proteins in double knockout (PAK1&4KO) compared with WT cells. Each heatmap represents the Z-scored $\log_2$ fold-change values of proteins identified by LC–MS proteomic analysis.

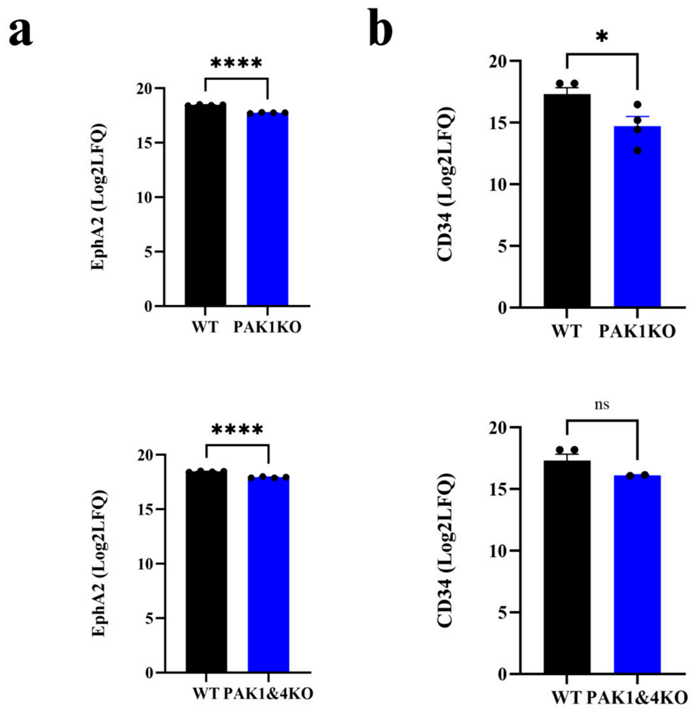

Figure S2. PAK1 and PAK1&4 knockout suppress vasculogenic mimicry-associated markers. Proteomic analysis revealed that PAK1KO and PAK1&4KO pancreatic cancer cells exhibited reduced expression of EphA2 (a) and CD34 (b), suggesting that loss of PAK1 may also impair vasculogenic mimicry. WT: wild type; KO: knockout; *P<0.05, ****P<0.0001; ns: not significant.
