# Supplementary figures and images for: The Effects of PAK-Regulated Tumour Vasculature on Gemcitabine Response of Pancreatic Cancer
Source: Cancers (Basel). 2025 Oct 26;17(21):3434. doi: 10.3390/cancers17213434 (PMC12607862; doi:10.3390/cancers17213434)

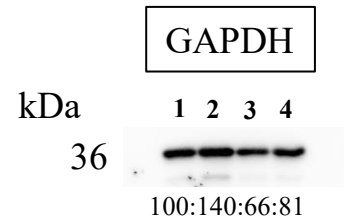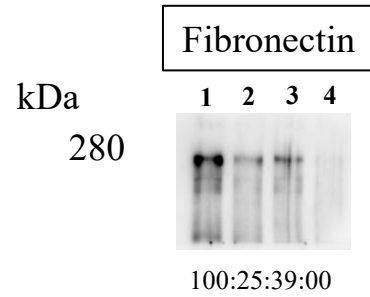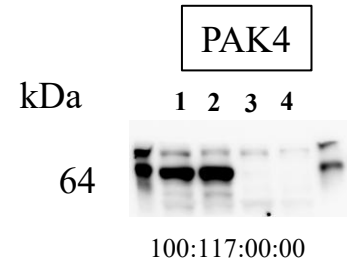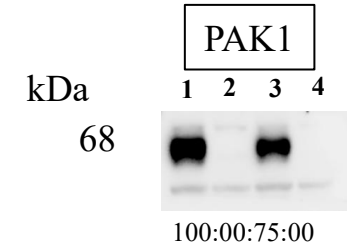

- (1) WT
- (2) PAK1KO
- (3) PAK4KO
- (4) PAK1&4KO

Figures 1 and 5

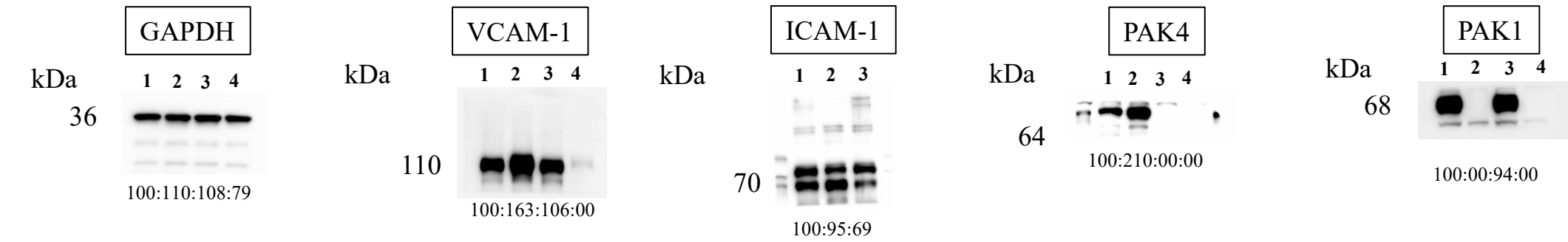

- (1) WT
- (2) PAK1KO
- (3) PAK4KO
- (4) PAK1&4KO

Figure 2

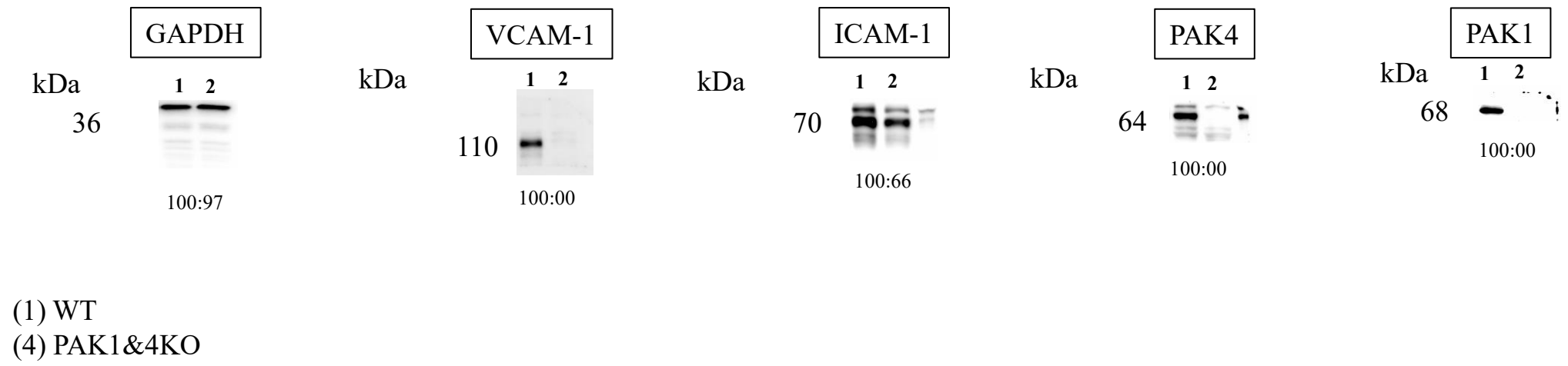

Figure 6

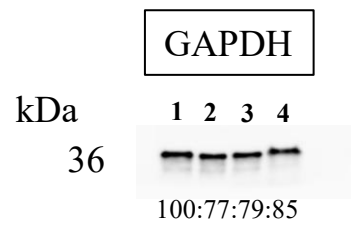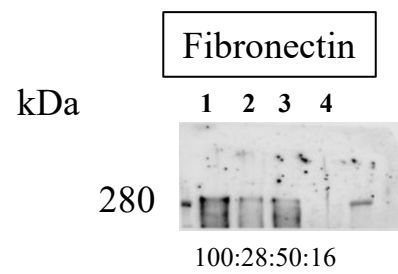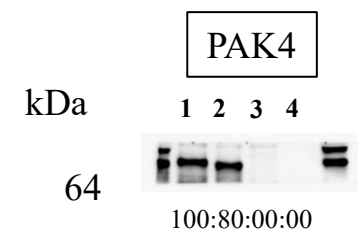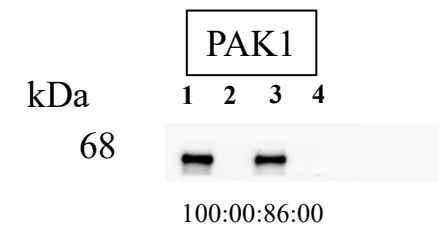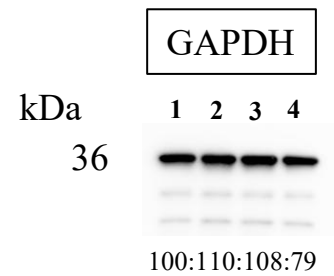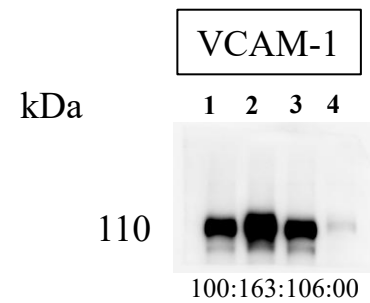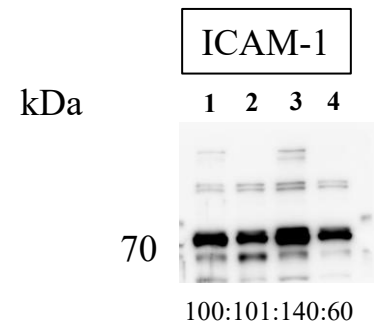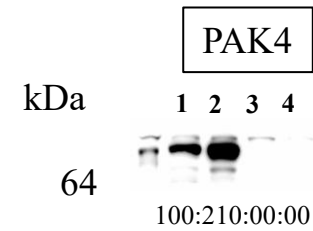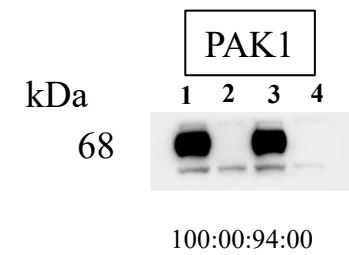

- (1) WT
- (2) PAK1KO
- (3) PAK4KO
- (4) PAK1&4KO

Supplement: Supplementary file 1 [file cancers-17-03434-s001.zip › File S1. Original Western blot figures.pdf]
